# The gelatinase biosynthesis‐activating pheromone binds and stabilises the FsrB membrane protein in *Enterococcus faecalis* quorum sensing

**DOI:** 10.1002/1873-3468.13634

**Published:** 2019-10-21

**Authors:** Sean Littlewood, Helena Tattersall, Charlotte S. Hughes, Rohanah Hussain, Pikyee Ma, Stephen E. Harding, Jiro Nakayama, Mary K. Phillips‐Jones

**Affiliations:** ^1^ School of Pharmacy and Biomedical Sciences University of Central Lancashire Preston UK; ^2^ Diamond Light Source Ltd. Harwell Science and Innovation Campus Didcot UK; ^3^ Astbury Centre for Structural Molecular Biology University of Leeds UK; ^4^ National Centre for Macromolecular Hydrodynamics School of Biosciences University of Nottingham Sutton Bonington UK; ^5^ Department of Bioscience and Biotechnology Faculty of Agriculture Graduate School Kyushu University Fukuoka Japan; ^6^ Present address: Paul Scherrer Institute Villigen Switzerland

**Keywords:** circular dichroism, *Enterococcus faecalis*, FsrB, quorum sensing

## Abstract

Quorum‐sensing mechanisms regulate gene expression in response to changing cell‐population density detected through pheromones. In *Enterococcus faecalis*, Fsr quorum sensing produces and responds to the gelatinase biosynthesis‐activating pheromone (GBAP). Here we establish that the enterococcal FsrB membrane protein has a direct role connected with GBAP by showing that GBAP binds to purified FsrB. Far‐UV CD measurements demonstrated a predominantly α‐helical protein exhibiting a small level of conformational flexibility. Fivefold (400 μm) GBAP stabilised FsrB (80 μm) secondary structure. FsrB thermal denaturation in the presence and absence of GBAP revealed melting temperatures of 70.1 and 60.8 °C, respectively, demonstrating GBAP interactions and increased thermal stability conferred by GBAP. Addition of GBAP also resulted in tertiary structural changes, confirming GBAP binding.

## Graphical abstract

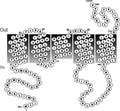

## Abbreviations

**DDM** *, n*‐dodecyl‐β‐d‐maltoside

**GBAP**, gelatinase biosynthesis‐activating pheromone

**GelE**, gelatinase

**IPTG**, isopropyl thiogalactoside

**PCA**, Principal component analysis

The FsrB membrane protein is a component of the Fsr quorum‐sensing system of the Gram‐positive opportunistic infection agent *Enterococcus faecalis* 1. The Fsr system comprises four components; in addition to FsrB (believed to process and transport the Fsr peptide pheromone across the membrane), these are FsrA (a response regulator), FsrD [a propeptide which ultimately becomes cleaved to form the peptide pheromone known as gelatinase biosynthesis‐activating pheromone (GBAP)] and FsrC (a histidine protein kinase; 2, encoded by the *fsrABDC* operon.

In *E. faecalis*, the Fsr system upregulates expression of gelatinase (GelE) and serine protease (SprE) virulence factors in a cell density‐dependent manner and in this regard resembles the much‐studied regulation of toxin synthesis by the *Staphylococcus aureus* Agr quorum‐sensing system 3, 4, 5, 6, 7, 8, 9, 10. Indeed, the Fsr system is the main activator of *gelE* expression 11, 12, 13, but has also been implicated in virulence independently of *gelE* 11. The peptide mediator of Fsr quorum sensing (GBAP) is a peptide lactone encoded by *fsrD* located just downstream of, and in frame with *fsrB* 14, 15. Mutations in either the Fsr system and/or in GelE production decrease the severity of disease in several model systems including a rabbit endophthalmitis model, *Arabidopsis thaliana*,* Caenorhabditis elegans*, mouse and an endocarditis model 13, 16, 17, 18, 19, 20. A deletion mutant of *fsrB* exhibited significantly reduced virulence in a rabbit endophthalmitis model, which was restored by complementation using an intact *fsrB* gene 16.

**Figure 1:**
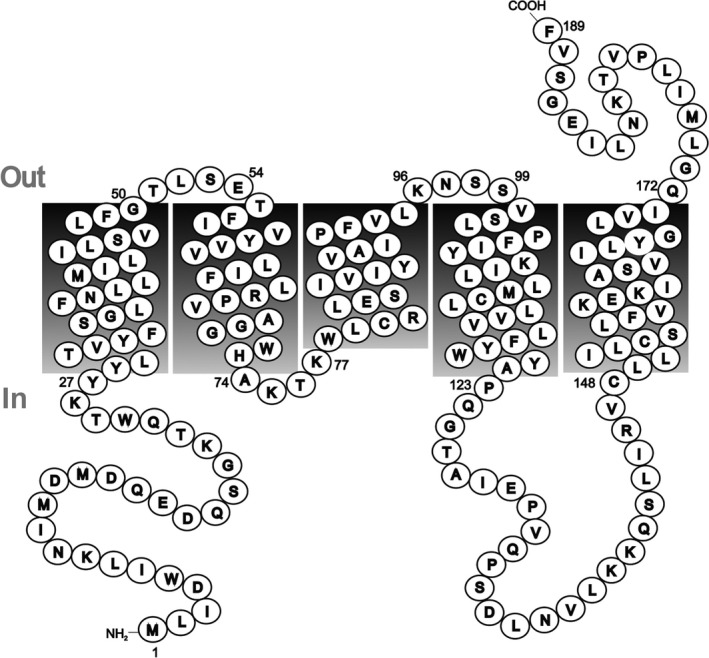
Schematic representation of FsrB. Transmembrane helices were predicted using the TMHMM membrane protein topology prediction method based on a hidden Markov model (https://www.expasy.org/proteomics). The predicted molecular mass is 21 591 Da (189 residues).

By analogy with the staphylococcal Agr quorum‐sensing system (and AgrB specifically), FsrB is predicted to serve both as a cysteine protease‐like enzyme responsible for post‐translational processing of the FsrD propeptide to give mature GBAP 15 and also possibly as a membrane transporter responsible for GBAP export across the cytoplasmic membrane to the external environment 14, 21. Evidence for a proteolytic role for FsrB towards GBAP comes from the study of Nakayama *et al*. 22 in which the GBAP propeptide (FsrD) disappeared in western blot analysis when FsrB’ (a truncated version of FsrB lacking the protein product of the in‐frame *fsrD* gene) was present, an activity that was inhibited by addition of inhibitor ambuic acid 22. Consistent with its suggested transporter role, FsrB is predicted to be a membrane protein possessing five putative transmembrane segments (Fig. 1). There are three regions of potential importance in a processing role, based on their predicted nonmembrane locations: a 27‐residue region (including fMet) at the intracellular N terminus, a cytoplasmic loop of 25 residues (residues 123–148) between helices 4 and 5 also located intracellularly and a 17‐residue portion located extracellularly (residues 172–189). More than one of these may potentially be involved in processing and even in transport functions.

However, with regard to FsrB role, there is no experimental evidence to confirm these predictions and findings. The present study was undertaken to investigate whether FsrB is indeed linked with the GBAP pheromone, as is the case for AgrB and the AgrD pheromone in the equivalent Agr system 23. If so, we would expect to see interactions between the two molecules. Here, we utilise CD spectroscopy to determine whether GBAP specifically interacts with purified FsrB, which would be consistent with either or both of the above roles. We confirm the binding of GBAP to FsrB and further show that FsrB‐GBAP ligand binding results in stabilisation of FsrB structural conformation. GBAP binding stabilised and exerted changes in both the secondary and tertiary structural conformations of FsrB. These results confirm a role for FsrB in GBAP functioning during quorum sensing.

## Materials and methods

### Production of purified FsrB

The *fsrB* gene (EF1821) of *E. faecalis* V583 was cloned into expression plasmid pTTQ18His 24 as described previously 25, 26. Briefly, primers FsrB‐F: 5′‐CCGGAATTCCCTAATCGATTGGATTCTAAAAAATATTATGG‐3′ and FsrB‐R: 5′‐AAAACTGCAGCTGCAAAAACACTTCCTTCAATTAAATTTTTTG‐3′ were used to amplify the *fsrB* gene from *E. faecalis* V583 genomic DNA using polymerase chain reaction. The *fsrD* gene encoding the GBAP pheromone itself, which occurs in‐frame with, and just downstream of *fsrB*, was omitted from the cloning 15. Following digestion with *Eco*RI and *Pst*I restriction enzymes, the isolated amplified fragment was ligated into *Eco*RI‐, *Pst*I‐digested pTTQ18His to give pTTQ18His‐FsrB in which *fsrB* is expressed as a recombinant protein (FsrB‐His_6_, henceforth referred to as FsrB), possessing a predicted MNSLID N‐terminal sequence (where M is the initiating fMet and NS are the first two amino acid residues and derived from pTTQ18His), and an additional AAGGRGSHHHHHH sequence at the C terminus of the expressed protein for purification. Purified plasmids were verified by DNA sequencing.

The plasmid pTTQ18His‐FsrB was transformed into *Escherichia coli* BL21 [DE3]. For preparation of purified FsrB, 6 L volumes of *E. coli* BL21 [DE3]/pTTQ18His‐FsrB were cultured at 37 °C in Luria–Bertani broth containing 100 μg·mL^−1^ carbenicillin as described previously 25, 27, 28. Expression of *fsrB* was induced using 0.2 mm isopropyl thiogalactoside (IPTG) and cultures were incubated at an optimised postinduction temperature of 33 °C for a further 3 h prior to harvesting. Following preparation of mixed membranes by the methods described in Ma *et al*. 25, membrane proteins were solubilised using 1% (w/v) *n*‐dodecyl‐β‐d‐maltoside (DDM). His‐tagged FsrB was purified from solubilised material by nickel affinity chromatography as described previously 25 using wash buffers containing 20 mm imidazole and elute buffers containing 200 mm imidazole, both of which contained 0.05% DDM detergent. Purified FsrB was buffer exchanged into 10 mm HEPES pH 7.9 containing 10% glycerol, 100 mm NaCl and 0.05% DDM (HGSD buffer) using Bio‐Rad (Watford, Herts., UK) Econo‐Pac 10DG size exclusion desalting columns and concentrated.

### Circular dichroism spectroscopy measurements

CD measurements were made using the nitrogen‐flushed instrument on the B23 Synchrotron Radiation CD Beamline at the Diamond Light Source, Oxfordshire, UK, as described previously 2, 26. Purified FsrB was prepared in HGSD buffer. GBAP or control solvent additions were accompanied by 20‐min incubation at 20 °C prior to obtaining spectral data. All measurements were made at 20 °C unless otherwise stated.

Far‐UV (180–260 nm) measurements employed 80 μm FsrB in HGSD buffer. GBAP dissolved in 30% acetonitrile was added to a final concentration of 400 μm to give fivefold excess of the pheromone (5 : 1 GBAP : FsrB). Equivalent concentrations of acetonitrile were added in control reactions; final concentrations of acetonitrile were 1.5%. Four scans were acquired using an integration time of 1 s, a path length of 0.2 mm and a slit width of 1.0 mm equivalent to 1.2 nm bandwidth.

Secondary structural composition was determined by analysis of the far‐UV spectral data using the CONTINLL algorithm of B23 beamline OLIS Globalworks software as described previously 2.

For thermal denaturation studies, spectral data for FsrB (80 μm) in the presence or absence of fivefold GBAP were collected at increasing temperatures from 20 to 95 °C, in 5 °C increments with 10‐min equilibration time followed by a return to 20 °C in one step of 30 (min) equilibration time.

Measurements in the near‐UV region (250–340 nm) were made in cells of 1 cm path length using 16 μm FsrB in HGSD buffer at 20 °C. To investigate the effect of fivefold molar excess GBAP, averaged data from 10 scans (integration time 1 s) were obtained for samples in the presence of either fivefold (80 μm) GBAP plus a final concentration of 0.84% acetonitrile, or 0.84% acetonitrile. All spectra of relevant background buffers, solvents, GBAP alone, etc., were subtracted from the experimental data to give difference spectra, which were then converted into mean residue ellipticity as described previously 2.

The mean residue weight for recombinant FsrB was taken to be 114. The cyclic GBAP pheromone (QN(SPNIFGQWM) used in the present study was prepared synthetically as described previously 14.

### SDS/polyacrylamide gel electrophoresis and western blotting

FsrB preparations were loaded onto SDS/polyacrylamide gels (5% stacking/12.5% resolving acrylamide: bisacrylamide gels) 29. Following electrophoretic separation, proteins were either stained with Coomassie blue 29 or transferred by electroblotting onto polyvinylidene fluoride membrane for western blotting with INDIA™ HisProbe‐horseradish peroxidase for detection of histidine‐tagged proteins, as described previously 25.

### Protein determination

FsrB concentrations were determined spectrophotometrically as described previously using a molar extinction coefficient, ε (FsrB‐His_6_), for recombinant FsrB of 41 410 m ^−1^·cm^−1^.

## Results

### Preparation of purified FsrB

**Figure 2:**
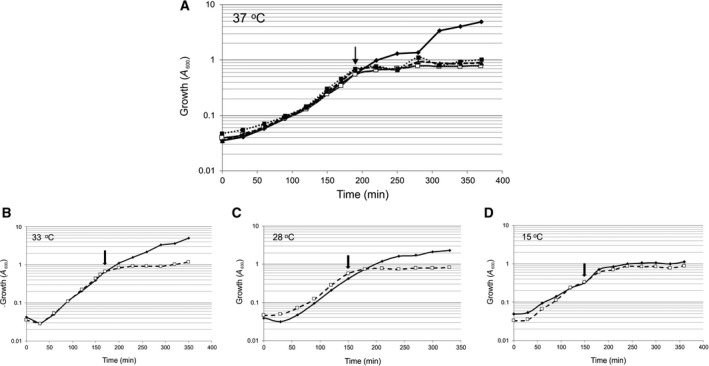
Optimisation of inducer and temperature conditions for culturing *E. coli* BL21 [DE3]/pTTQ18His‐FsrB in LB medium containing 100 μg·mL^−1^ carbenicillin. (A) Effect of IPTG concentrations on growth (absorbance at 600 nm, *A*_600_): no IPTG (solid black line, ♦); 0.2 mm IPTG (grey line, □), 0.5 mm IPTG (dashed line, ▲); and 1.0 mm IPTG (dotted line, ■). Growth was at 37 °C; the arrow indicates point of IPTG addition. Panels (B–D) show growth at postinduction temperatures of 33, 28 and 15 °C, respectively, following culturing at 37 °C in LB selective broth and addition of 0.2 mm IPTG indicated by the arrows. No IPTG addition (solid black line, ♦); 0.2 mm IPTG, (dashed line, □).

Following verification of the *fsrB* gene in the expression plasmid pTTQ18His‐FsrB by sequencing, the plasmid was transformed into the expression host *E. coli* BL21 [DE3] which was cultured in selective Luria–Bertani (LB) medium as described in Materials and Methods and induced for FsrB production using 0.2 mm IPTG followed by 3‐h incubation at 33 °C prior to harvesting. IPTG induction resulted in an almost immediate cessation in growth compared with cultures lacking IPTG addition (Fig. 2), indicative of successful FsrB expression due to FsrB insertion into *E. coli* membranes resulting in deleterious effects on *E. coli* growth, as commonly observed during membrane protein expression 30, 31.

**Figure 3:**
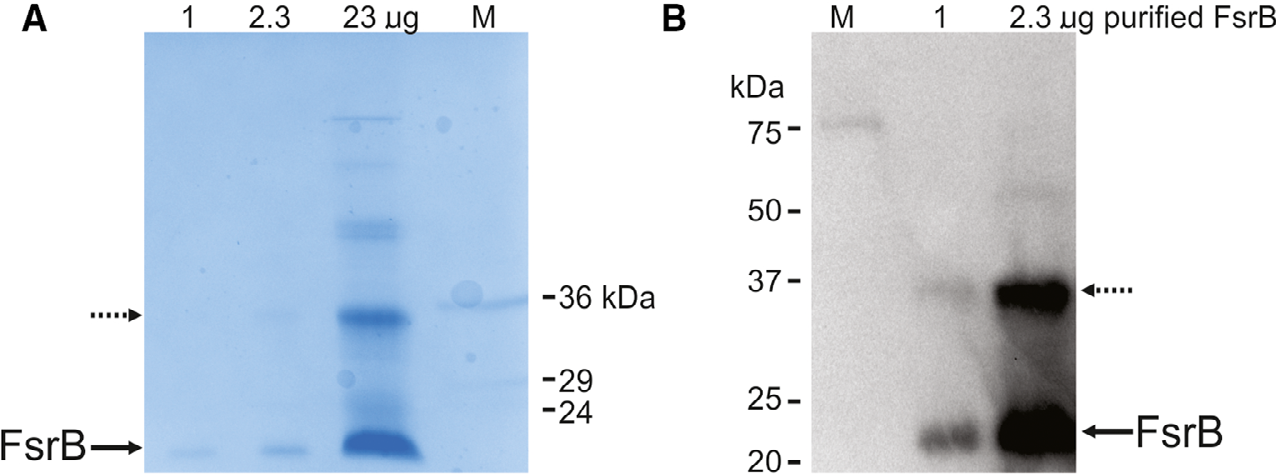
Production and confirmation of purified His‐tagged *E. faecalis* FsrB. (A) SDS/polyacrylamide gel (5% stacking and 12.5% resolving gel) of purified intact FsrB (1, 2.3 and 23 μg) (predicted molar mass 23.2 kDa) visualised using Coomassie blue staining. M, molecular mass markers; and (B) western blot using 1 and 2.3 μg purified FsrB with an INDIA His probe to detect the presence of the C‐terminal hexahistidine tag. Arrows denote the positions of the monomeric (solid arrow) and minor levels of multimeric (dashed arrow) intact protein.

Purification of FsrB *via* the hexa‐Histidine tag engineered at the C terminus of the protein was achieved using well‐established methods developed in our laboratory for members of a range of membrane protein classes 25, 26, 27, 28). Figure 3 summarises the successful application of these methods for production of FsrB. In SDS/polyacrylamide gels, the protein exhibits an apparent molar mass of ~ 22 kDa (Fig. 3A), which is in good agreement with the calculated mass of 23.2 kDa for the His‐tagged protein. The additional minor band of apparent mass ~ 35 kDa corresponds to a multimeric form of FsrB, as commonly observed for other purified membrane proteins during SDS/PAGE analysis 32. Both 22‐ and 35‐kDa bands gave positive signals in the western blot confirming the presence of the hexa‐Histidine tag (Fig. 3B).

### Investigations of FsrB secondary structural conformation with and without GBAP

**Figure 4:**
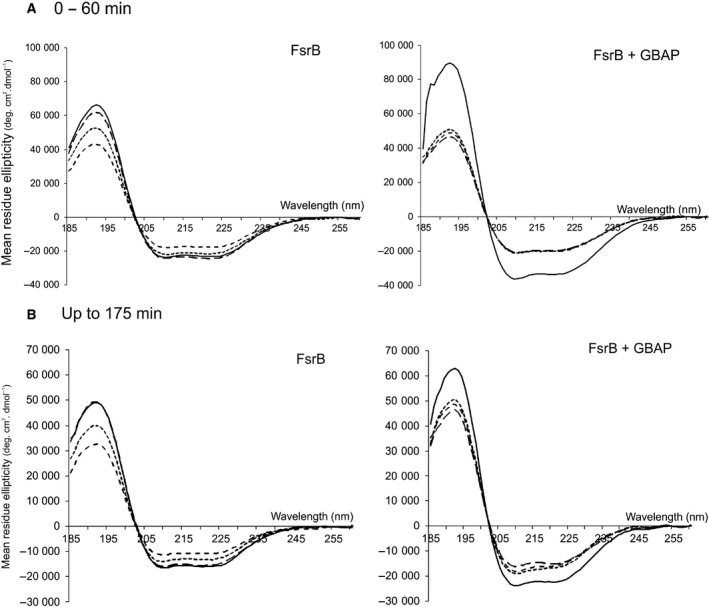
Far‐UV difference spectra of FsrB (80 μm) in the presence and absence of fivefold (400 μm) GBAP, and suspended in 10 mm HEPES pH 7.9, 10% glycerol, 100 mm NaCl and 0.05% DDM. Contributions by buffer and acetonitrile (used to dissolve GBAP) were removed by subtracting the relevant control spectra. Acetonitrile concentrations did not exceed 1.5%. Data are average of four scans. Increment 1 nm, path length 0.2 mm and slit width 1 mm, using loading volumes of 5 μL. Spectra were obtained 20 min after GBAP or acetonitrile addition (time 0, shown by the solid black lines and at (A) 10‐, 50‐ and 60‐min intervals thereafter for FsrB (long dashed line, dashed line and dotted line, respectively) and at 40‐, 50‐ and 60‐min intervals thereafter for FsrB + GBAP (long dashed line, dashed line and dotted lines, respectively); and (B) in a repeat experiment at 39, 78 and 130 min thereafter for FsrB (long dashed line, dashed line and dotted line, respectively) or at 35, 110 and 175 min thereafter for FsrB + GBAP (long dashed line, dashed line and dotted line, respectively in both cases).

Retention of secondary structural integrity of the FsrB protein postpurification was investigated by CD spectroscopy measurements in the far‐UV region (185–260 nm). Purified FsrB produced spectra with negative minima at 210 and 224 nm and a strong positive spectral peak at 193 nm characteristic of a prevailing α‐helical structure 33, 34 and thus confirming the integrity of the heterologously produced protein. Interestingly, despite the presence of DDM detergent at a concentration of 0.05% which is well above the CMC value of this detergent (~ 0.17 mm, 0.0087%) 35, and which stabilises other membrane proteins such as FsrC in DDM micelles with regard to obtaining reliable and reproducible CD spectra 2, FsrB alone produced difference spectra of increasingly shallow characteristics over a 60‐min period, indicative of a small but detectable lack of stability in the secondary structural conformation of FsrB (Fig. 4A). By contrast, in the presence of fivefold GBAP, difference spectra were transiently more intense (more positive at 193 nm and more negative at 210 and 214 nm) at time 0, indicative of higher secondary structural composition in the initial presence of the pheromone, followed by shallower but stable overlayable spectra indicative of stable secondary structure conformation (Fig. 4A). All these effects were reproducible, and Fig. 4B shows an example of a repeat experiment, this time over a 175‐min period in which the same trend was observed (Fig. 4B).

Analysis of the secondary structural composition using the CONTINLL algorithm reveal a significantly higher proportion of α‐helical content initially upon addition of GBAP [increased from 61.0 (±1.8) to 75.1 (±2.2)% total α‐helix], accompanied by lower % β‐strand and unordered content [6.3–16.7 (±9.0) to 2.2–5.9 (±3.0) % β‐strand; and 17.7–26.8 (±9.0) to 14.4–22.9 (±3.0) % unordered structure] (Table 1). With time, shallowing of difference spectra was again observed (Fig. 4), but unlike the spectra of FsrB alone, the presence of GBAP resulted in difference spectra that were stable and could be reliably overlaid within 40 min suggesting that GBAP may have a stabilising effect on FsrB (and therefore possibly indicative of GBAP binding to FsrB). The data in Table 1 suggest that after 2 h, there remain small but statistically nonsignificant differences in the α‐helical and β‐strand contents of the protein in the presence and absence of the GBAP ligand, with values of 61.3–64.3 ± 9.0% α‐helix and 4.4–6.3 ± 9.0% β‐strand (Table 1).

**Table 1:** Secondary structure composition of FsrB in DDM micelles using the mean values derived from the CONTINLL algorithm. Data derived using the SMP50 and SP37 + 13 membrane protein datasets. Average of four replicates. GBAP was added at a five‐fold excess

Scan	H1: Alpha helix	H2: Distorted alpha helix	Alpha helix (both types)	S1: Beta strand	S2: Distorted beta strand	Beta strand (both types)	T: Turns	U: Unordered	Standard deviation
FsrB									
0 min	42.1	18.8	61.0	4.7	3.8	8.5	12.8	17.7	1.8
39 min	44.4	18.9	63.3	5.1	3.0	8.1	9.3	19.3	1.5
78 min	34.1	14.8	48.9	11.2	5.4	16.7	7.7	26.8	4.3
130 min	40.4	20.9	61.3	4.5	1.9	6.3	8.3	24.3	9.0
FsrB + GBAP									
0 min	53.8	21.2	75.1	0.8	1.4	2.2	8.3	14.4	2.2
35 min	41.0	18.7	59.8	2.9	3.1	5.9	11.4	22.9	1.7
110 min	42.0	18.4	60.4	1.4	3.5	5.0	12.2	22.4	1.7
175 min	45.5	18.8	64.3	2.2	2.2	4.4	9.9	21.3	3.0

**Figure 5:**
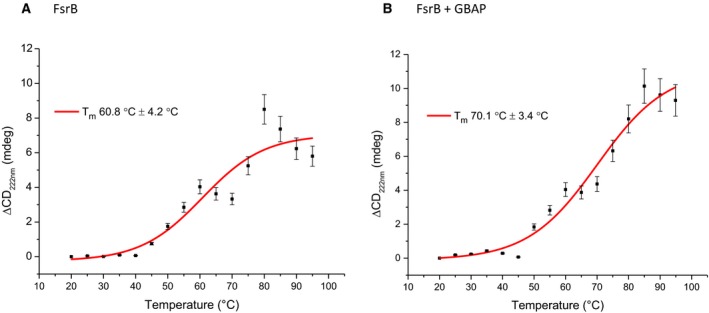
Thermal denaturation of FsrB (80 μm) in the (A) absence and (B) presence of fivefold (400 μm) GBAP. FsrB protein was incubated at 20 °C for 20 min. The temperature was then increased in 5 °C increments to 95 °C and maintained at each temperature for 5 min prior to acquiring spectral data in the 180–260 nm range. Δmdeg changes at 222 nm were plotted against temperature and curves fitted to a sigmoidal function using CDApps. Calculated melting temperatures are shown. Circled markers indicate the Δmdeg following a return to 20 °C after the temperature ramp. Data are the average of four scans.

To investigate further the possibility that the presence of GBAP results in a more stable FsrB conformation, thermal denaturation experiments were undertaken in which CD spectra were acquired during temperature ramping of FsrB (in the presence or absence of GBAP), in 5 °C successive increases from 20 to 95 °C, before being returned to 20 °C once more. Four replicate spectra were obtained at each temperature. The changes in ellipticity (at 222 nm) measured as a function of temperature are shown in Fig. 5. Melting temperatures of 60.8 ± 4.2 °C for FsrB, and the higher value of 70.1 ± 3.4 °C for FsrB in the presence of GBAP were obtained, demonstrating that the presence of GBAP does indeed result in a marked increased stability of FsrB. Principal component analysis (PCA) undertaken as described previously 36 for the thermal melt of FsrB alone and in the presence of GBAP reveals a transition at 50 °C into two distinct species (components) in both cases representing 75.7% variance (Fig. S1). From the PCA loading graph, the route of transition taken by the proteins in the two conditions is not too dissimilar, as demonstrated by the grouping of the variances at each temperature for both conditions.

Upon return to 20 °C, there was no significant further change in the spectrum at 222 nm, suggesting that the temperature‐induced denaturation/changes in FsrB conformation were irreversible under these conditions, whether GBAP is present or not (data not shown).

### Investigations of FsrB tertiary structural conformation with and without GBAP

To determine whether GBAP binding affects the tertiary structural conformation of FsrB and/or GBAP itself, CD measurements were undertaken in the near‐UV region which reports changes due to ligand binding in the environments of the Phe (broadly, 250–270 nm), Tyr (270–290 nm), Trp (280–300 nm) residues and any disulphide bonds (broad weak signals across the near‐UV spectrum) 37. FsrB is predicted to possess 5 Trp, 9 Tyr, 10 Phe and 4 Cys residues.

**Figure 6:**
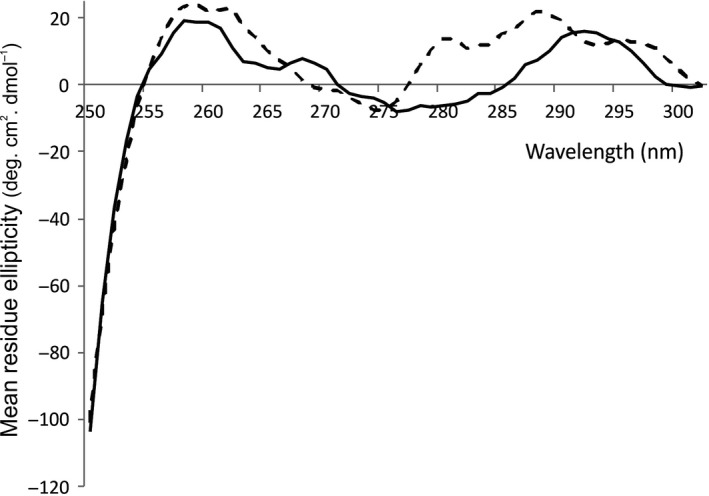
Near‐UV difference spectra of FsrB (16 μm) in the absence (solid black line) and presence (dashed black line) of fivefold GBAP (80 μm), and suspended in 10 mm HEPES pH 7.9, 10% glycerol, 100 mm NaCl and 0.05% DDM. Contributions by buffers and acetonitrile (used to dissolve GBAP) were removed by subtracting relevant control spectra. Data are average of 10 scans. Increment 1 nm, path length 10 mm and slit width 1 mm, using reaction volumes of 70 μL. Spectra were obtained 20 min after GBAP or acetonitrile addition. Spectra were set to zero at 302 nm.

Figure 6 shows the near‐UV difference spectra for FsrB in the presence and absence of fivefold GBAP, corrected for any contributions arising from GBAP itself, buffer, detergent or acetonitrile solvent. Any changes observed in the spectra in the presence of GBAP could be due to changes in FsrB and/or in GBAP itself upon interaction. There is no obvious evidence of any disulphides present [in principle both acetonitrile and GBAP (the latter possessing a lactone link joining Ser3 and Met11 in this cyclic molecule), are oxidising molecules], as the spectra overall overlay reasonably well.

The FsrB spectrum reveals a strong region of Phe residues peaking at ~ 259 and 268 nm (Fig. 6). The corresponding region for the FsrB + GBAP spectrum reveals one discrete larger positive spectral peak in the 250–270 nm Phe region, thus possibly suggesting changes in the environments of some Phe residues in the presence of the ligand. The FsrB spectrum also exhibits a distinct trough in the 270–285 nm region corresponding to Tyr and Trp residues, with a spectral peak occurring at ~ 292 nm which might correspond more to Trp than Tyr residues at this longer wavelength. The corresponding region of the FsrB + GBAP spectrum exhibits three strongly positive spectral peaks with maxima at approximately 280, 289 and 297 nm, indicative of GBAP interactions affecting the environments of Trp and Tyr residues in FsrB and/or GBAP (Fig. 6).

Importantly, taken together these data show that GBAP binds to purified recombinant FsrB and also that as a result of binding by this ligand, the secondary structural conformation of FsrB acquires increased stability (Figs 4 and 5; Table 1) and the tertiary structure of FsrB and/or GBAP is changed, evidenced by the changed spectra in the 270–297 nm Tyr/Trp region and in the 250–270 nm region of Phe residues (Fig. 6).

## Discussion

In the present study, FsrB was successfully expressed and purified from *E. coli* membranes as a Histidine‐tagged protein. Induction of expression in the *E. coli* BL21 [DE3] host was accompanied by an almost immediate cessation of exponential growth (Fig. 2), as observed during the expression of many membrane proteins, presumably because packing of the overexpressed heterologous protein into *E. coli* membranes is deleterious to growth. Following successfully solubilisation from *E. coli* membranes and purification of the protein into detergent micelles, FsrB was shown here to retain structural integrity as demonstrated by the presence of secondary structure in CD measurements made in the far‐UV region (Fig. 4). From these measurements, it is also clear that the protein exhibits flexibility though small as depicted by CD spectroscopy as a function of time (Fig. 4A). Under the detergent conditions used here [using concentrations (0.05%) well above that of the CMC value], it is unlikely that instabilities of FsrB conformation would arise as a result of insufficient detergent as we have observed previously for FsrC when lower (0.025%) detergent levels were used 2. It is possible that the protein requires native membrane to minimise flexibility; however, we have shown in the present study that the presence of the membrane environment is not required to observe GBAP binding to FsrB, which results in consistent spectra and increased stability of FsrB secondary structure upon GBAP addition (following an initial transient increase in secondary structural composition). It is also possible that some UV denaturation of FsrB occurs as spectra are acquired accounting for the flexibility/instability observed, or even migration of the protein within the detergent micelle; if so, these explanations nonetheless remain consistent with the idea that GBAP binding reduces any such effects. The presence of GBAP resulted in an increase of almost 10 °C in the *T*_M_ value for FsrB and significant changes in tertiary structural conformation upon GBAP binding (Figs. 5 and 6). All these data therefore clearly demonstrate that FsrB binds GBAP and therefore that FsrB is likely to have a role in GBAP functioning during quorum sensing.

It is well established that ligand binding to proteins can increase or decrease protein thermal and/or conformational stability, and therefore, it seems reasonable to conclude from these results that GBAP binding is an example of a ligand that increases both such stabilities in FsrB. However, it is also possible that purified FsrB acquires a small degree of disorder postpurification, possibly through lack of the membrane environment, and that the disorder particularly at the tertiary structural level is relieved upon ligand binding 38.

Further studies are required to determine the precise role(s) of FsrB in the quorum‐sensing process. Now that it has been shown that GBAP binds to FsrB (through CD spectroscopy), it seems probable that such roles could involve direct FsrB‐GBAP interactions such as post‐translational processing of the newly synthesised GBAP and/or export of GBAP across the enterococcal membrane to the cell exterior, as suggested by others previously. Indeed, proteolytic activity towards FsrD (pro‐GBAP) has been observed 22. The corresponding staphylococcal quorum‐sensing protein AgrB is also believed to be involved in similar processes 3, 21, 39, and again proteolytic processing of the equivalent AgrD cyclic pheromone has been demonstrated 40. In the case of both FsrB and AgrB, possible additional roles for these proteins in peptide pheromone export across the membrane have yet to be investigated. PSI‐BLAST searches of each of the nonmembrane spanning regions predicted for FsrB reveal (not unexpectedly) similarity with many protein domain types but it is interesting that residues 123–148 exhibits 69% identity (75% similarity) in 16 residues to several ABC transporter substrate‐binding proteins, whilst residues 172–189 exhibit 65% identity (70% similarity) in 17 residues to eukaryotic K(+) efflux antiporter 3 proteins. Future *in vitro* mutagenesis studies of key residues may resolve whether these similarities are indeed of significance, and future studies of ligand binding strengths may also be informative.

## Author contributions

SL, PM and HT performed the research; CSH, SL, SHE, RH and MP‐J analysed data and interpreted results; JN and MP‐J designed the study and MP‐J wrote the manuscript.

## Supporting information


**Fig. S1.** Plots for principal component analysis (PCA) (A) loading and (B) PCA screening.
