# Supplementary material for: The gelatinase biosynthesis‐activating pheromone binds and stabilises the FsrB membrane protein in Enterococcus faecalis quorum sensing
Source: FEBS Lett. 2019 Oct 21;594(3):553–63. doi: 10.1002/1873-3468.13634 (PMC7028047; doi:10.1002/1873-3468.13634)
Supplement: Supplementary file 1 — Fig. S1. Plots for principal component analysis (PCA) (A) loading and (B) PCA screening. [file FEB2-594-553-s001.doc]

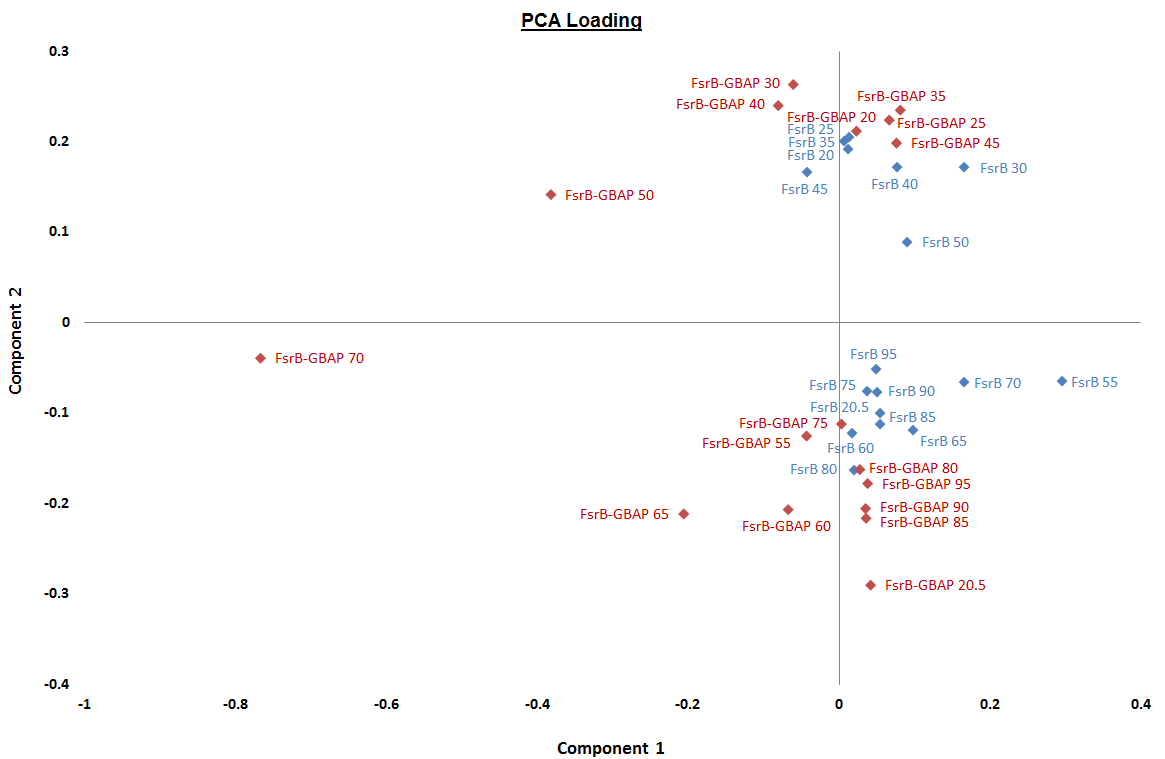


**A.**

**B.**


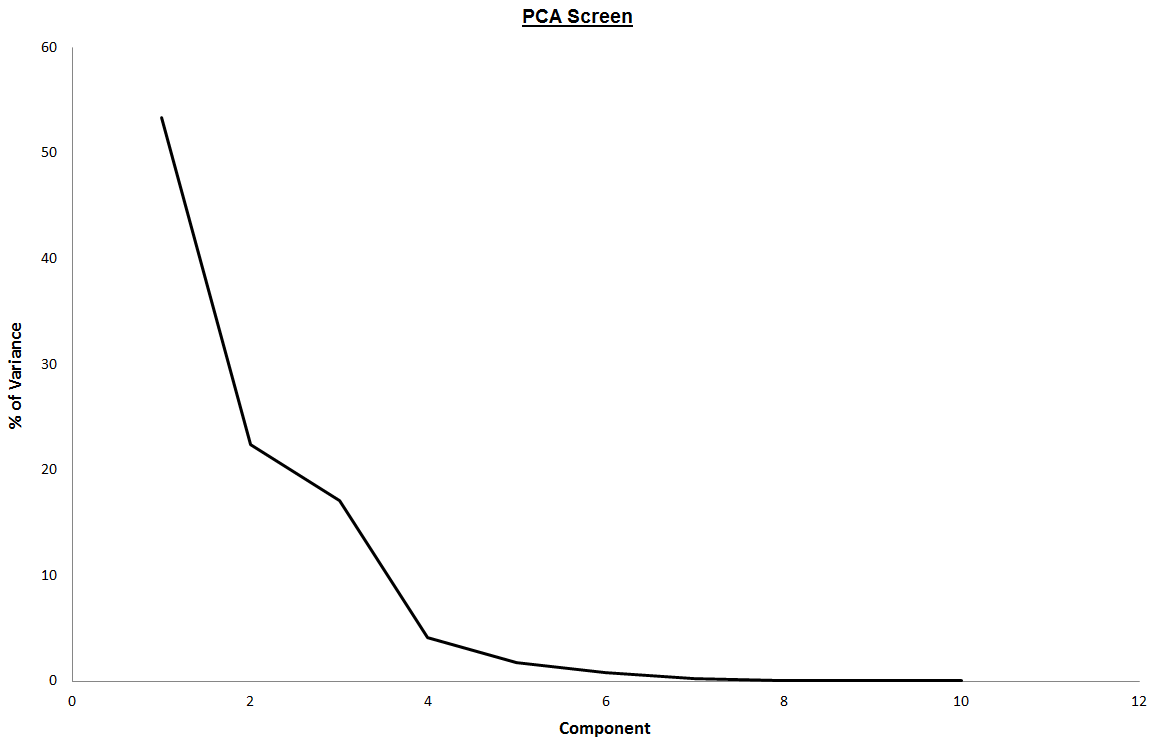


**Fig. S1.** Plots for principal component analysis (PCA) (A) loading and (B) PCA screening. PCA analysed from CD spectra of thermal melt experiments using CDApps [36]. Blue, FsrB; red, FsrB + GBAP.
